# Unmasking primary thyroid tuberculosis - A rare but deceptive diagnosis: A case report with review of literature

**DOI:** 10.1016/j.ijscr.2025.111201

**Published:** 2025-03-25

**Authors:** Areeba Khursheed, Shahbaz Habib Faridi, SyedHasan Harris, Bushra Siddiqui, Mohammad Nafees Ahmad, Ezaz Ahmed

**Affiliations:** a Department of Surgery, JN Medical College, Aligarh Muslim University, Aligarh, Uttar Pradesh, India; b Department of Pathology, JN Medical College, Aligarh Muslim University, Aligarh, Uttar Pradesh, India

**Keywords:** Case report, Thyroid tuberculosis, ESR, Tuberculin test, Lymphohistiocytic cluster

## Abstract

**Introduction and importance:**

Thyroid tuberculosis is a rare condition, even in regions where tuberculosis (TB) is more common, possibly due to the thyroid gland's relative immunity. Diagnosing thyroid TB can be challenging as the clinical signs are often nonspecific.

**Case presentation:**

A 75-year-old male presented with a swelling in the midline of the neck with an abscess in the lower portion of the swelling. Thyroid function tests were normal, and the tuberculin skin test returned positive. USG of the neck showed heterogeneous hypoechoic collection in the left lobe of thyroid, with surrounding inflammation and multiple subcentimetric lymph nodes in right upper, mid and lower jugular region showing necrosis. FNA revealed mixed inflammatory infiltrate predominantly neutrophils with lympho-histiocytic clusters in necrotic background. No signs of tuberculosis were detected in other organs. Based on these findings, primary thyroid tuberculosis was diagnosed. The patient was started on anti tubercular treatment resulting in a favorable clinical outcome.

**Clinical discussion:**

TB rarely presents as a thyroid mass. A history of prior tuberculosis, the presence of cervical lymphadenopathy, and an elevated erythrocyte sedimentation rate (ESR) can support the diagnosis. Fine-needle aspiration cytology (FNAC) is an effective diagnostic method.

**Conclusion:**

Although rare, tuberculosis of the thyroid must be considered before deciding on a surgical management for disorders of the thyroid. Treatment typically involves anti-tuberculosis medications, but drainage may be needed for large abscesses, and thyroidectomy might be required if the FNAC results are inconclusive.

## Highlights

• Thyroid tuberculosis is a rare condition(0.1-0.4% of all thyroid diseases)
• The clinical manifestations of thyroid TB can vary significantly
• Due to the potential for misdiagnosis as a malignancy or other benign conditions, maintaining a high index of suspicion is essential to avoid unnecessary treatments
• Treatment typically involves anti-tuberculosis medications, while surgery is reserved for cases requiring drainage of large thyroid abscesses or partial thyroidectomy for significant gland involvement.

## 1 Introduction

Thyroid tuberculosis (TB) is an uncommon condition, even in regions where TB is endemic, accounting for approximately 0.1–0.4 % of all thyroid diseases [1]. Primary involvement of the thyroid by TB is even rarer.

Historically, the thyroid was considered resistant to TB until Lebert's report in 1862, where he described thyroid involvement in disseminated tuberculosis [2]. This rare occurrence is thought to be due to the thyroid's relative immune resistance.

The clinical manifestations of thyroid TB can vary significantly, ranging from asymptomatic to symptoms that may resemble benign or malignant thyroid disorders.

The progression of the disease can also be highly variable, influenced by factors such as thyroid dysfunction and the presence of complications.

While fine-needle aspiration cytology (FNAC) can be used to diagnose thyroid TB, its diagnostic yield is often low. A definitive diagnosis is typically made through histopathological examination, which shows caseating granulomas. Acid-fast bacillus (AFB) staining provides additional support for confirming TB. Due to the potential for misdiagnosis as a malignancy or other benign conditions, maintaining a high index of suspicion is essential to avoid unnecessary treatments, such as total thyroidectomy.

## 2 Case report

**Fig. 1:**
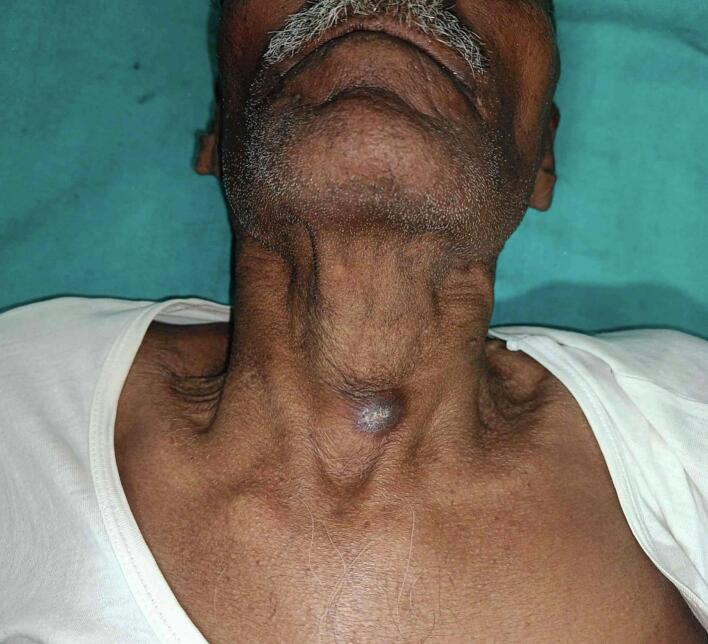
Clinical photograph showing thyroid swelling with healed abscess.

A 75-year-old male from eastern India presented to the outpatient clinic with a midline neck swelling that had been present for four months, accompanied by an abscess in the lower left part of the swelling for the past three months (Fig. 1). The patient did not have any prior history of BCG vaccination. Upon physical examination, a 3 × 3 cm firm, irregular swelling was noted in the midline, with minimal tenderness. The swelling moved with swallowing, and an abscess was observed in the lower left portion of the swelling. No palpable cervical lymphadenopathy was noted, and indirect laryngoscopy revealed no abnormalities.

Blood investigations were done which included a complete blood count, renal function and liver function tests, random blood sugars and serum electrolytes which were normal except for a markedly elevated erythrocyte sedimentation rate (118 mm/h). Thyroid function tests indicated a euthyroid state.

**Fig. 2:**
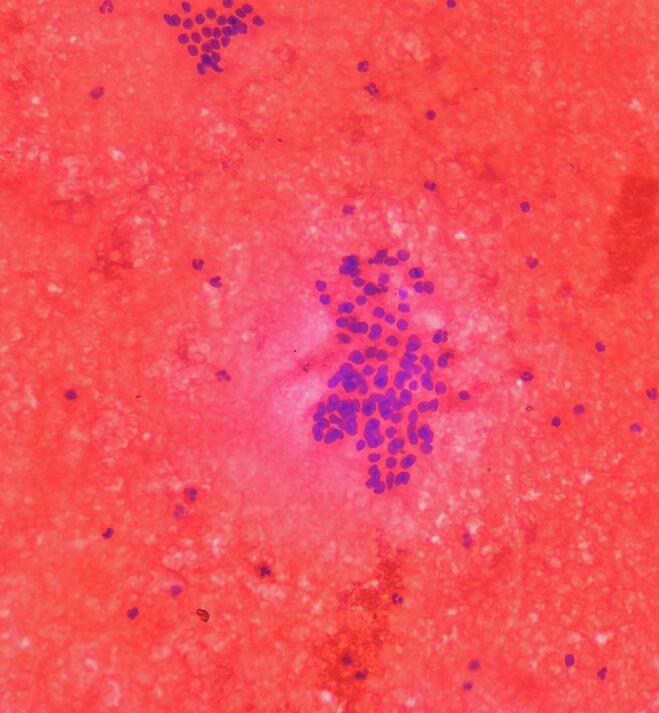
Fine need aspiration cytology from the left lobe of the thyroid gland showing thyroid follicular cells with blood mixed colloid in the background along with mixed inflammatory infiltrates.

**Fig. 3:**
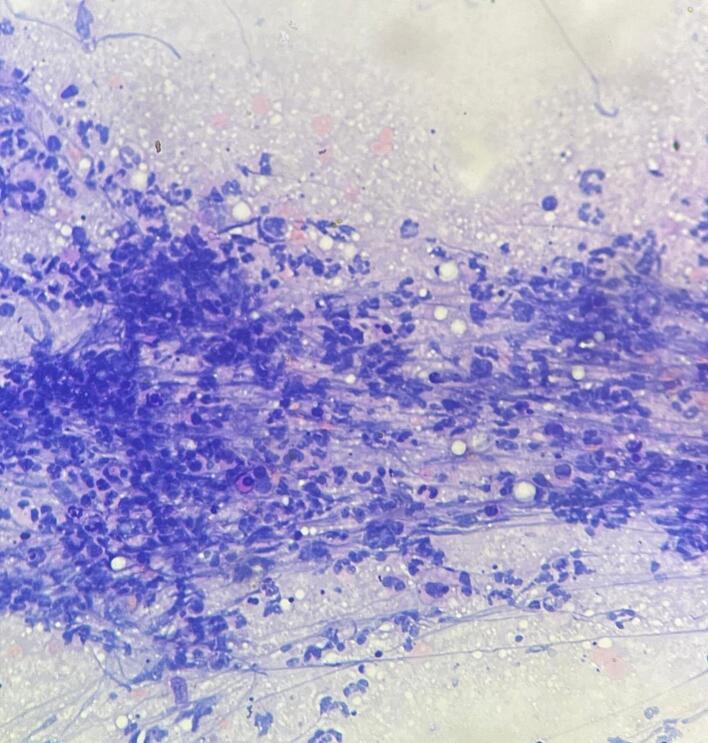
Fine need aspiration cytology from the thyroid abscess showing degenerated and viable neutrophils with lymphohistiocytes in the necrotic background (H&E ×100).

Fine needle aspiration cytology (FNAC) was performed from two distinct areas: (1) the enlarged left thyroid lobe and (2) the base of the abscess. The aspirate from the thyroid contained blood-mixed material, while the abscess yielded a whitish substance. Cytological examination showed normal follicular cells arranged in acinar and papillary formations from the thyroid (Fig. 2) and neutrophils with lympho-histiocytes in necrotic background suggestive of tuberculous etiology, likely of tuberculous origin (Fig. 3).

**Fig. 4:**
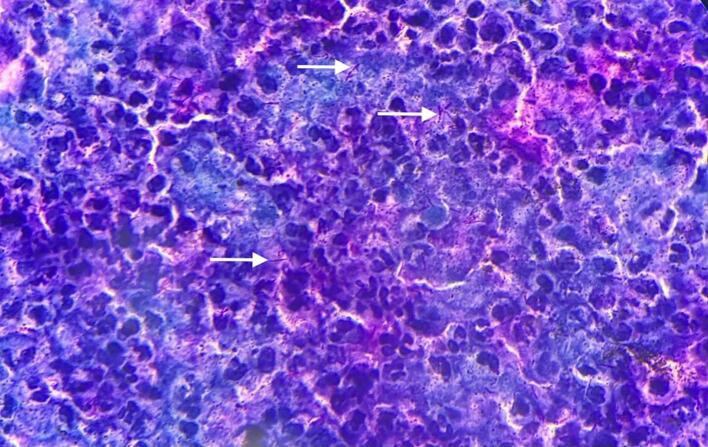
Ziehl-Neelsen stain ×100 showing acid fast bacilli (white arrows) and dense inflammatory infiltrates.

**Fig. 5:**
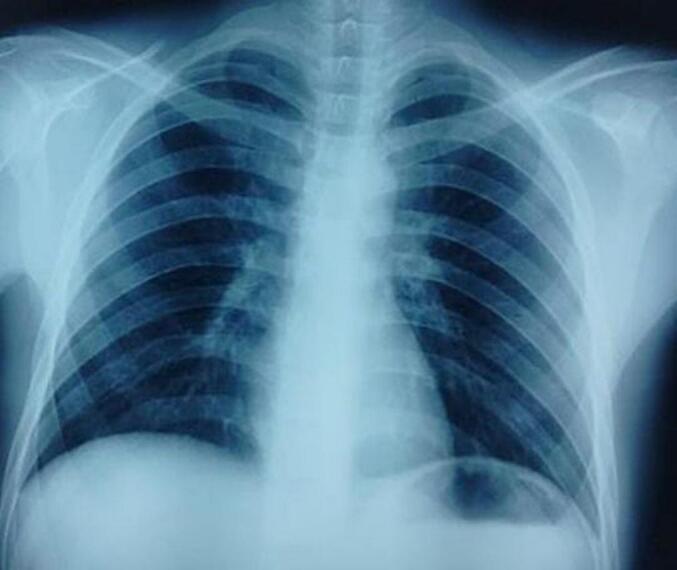
Chest x-ray of the patient showing no significant findings.

Pus from the abscess was sent for bacteriological culture and Ziehl-Neelsen staining for acid-fast bacilli (AFB), which revealed a high presence of AFB (Fig. 4). X-ray of the chest revealed no abnormalities (Fig. 5). Neck ultrasound revealed a heterogeneous hypoechoic collection (22 × 10 mm) in the left lobe of thyroid, surrounded by inflammation. Multiple small necrotic lymph nodes were observed in the right upper, middle, and lower jugular regions.

A Mantoux test using 1 TU of purified protein derivative (PPD) showed a positive result with an induration of 18 mm after 48 h. Other routine investigations, including chest x-ray, USG of the abdomen did not reveal any primary foci of tuberculosis. The patient was started on anti-tubercular therapy with two months of intensive phase (HRZE) followed by four months of continuous phase (HRE). The patient showed complete resolution of symptoms after a six months follow up.

## 3 Discussion

Tuberculosis of the thyroid gland is an uncommon condition, accounting for only 0.1–0.4 % of all thyroid diseases [1], with primary thyroid tuberculosis being even rarer. This low incidence is attributed to factors such as the gland's high iodine content, the antibacterial properties of colloid, its rich blood supply, the protective thyroid capsule, and the potential antitubercular effect of thyroid hormones [3]. The infection can spread to the thyroid either through hematogenous or lymphatic routes from a distant tuberculosis focus [4].

Barnes and Weatherstone identified two primary forms of thyroid tuberculosis: (1) miliary tuberculosis spread to the thyroid, often from pulmonary or cervical lymph node involvement, and (2) primary thyroid tuberculosis with no identifiable external focus [5].

The presentation of thyroid tuberculosis can vary greatly, ranging from asymptomatic cases to more severe manifestations like multiple diffuse lesions, goiter with caseating necrosis, cold abscesses, acute abscess formation, or chronic fibrosing tuberculosis [6].

In the early stages, thyroid function may remain normal, but later on, patients may develop hypo- or hyperthyroidism [7]. Routine lab tests are typically unremarkable, except for an elevated erythrocyte sedimentation rate. Imaging studies, such as chest X-rays and ultrasound of the abdomen and spine, can help locate any primary tuberculosis focus.

Seed (1944) outlined three essential criteria for diagnosing thyroid tuberculosis [1]:

1. Identification of acid-fast bacilli (AFB) in the thyroid tissue.
2. Presence of necrosis or abscess formation within the thyroid.
3. Evidence of a tuberculosis focus outside the thyroid.

Histological and bacteriological evidence is sufficient for diagnosis, although the third criterion is not mandatory when diagnosing primary thyroid tuberculosis.

Fine needle aspiration cytology (FNAC) under ultrasound guidance and histopathological analysis are considered the gold standard for diagnosing thyroid tuberculosis. FNAC can confirm the diagnosis in up to 73 % of cases [8]. The cytological patterns of tuberculous thyroiditis are categorized into three types [1]:

• Type I: Epithelioid granuloma without necrosis,
• Type II: Epithelioid granuloma with necrosis,
• Type III: Necrosis without granuloma.

Treatment typically involves anti-tuberculosis medications, while surgery is reserved for cases requiring drainage of large thyroid abscesses or partial thyroidectomy for significant gland involvement.

## 4 Conclusion

Although thyroid tuberculosis is uncommon, it should be considered as a potential diagnosis for thyroid masses, especially in regions where tuberculosis is prevalent. A history of prior tuberculosis, the presence of cervical lymphadenopathy, and an elevated erythrocyte sedimentation rate (ESR) can support the diagnosis, but thyroid tuberculosis can still occur without these signs. Fine-needle aspiration cytology (FNAC) is an effective diagnostic method. Treatment typically involves anti-tuberculosis medications, but drainage may be needed for large abscesses, and thyroidectomy might be required if the FNAC results are inconclusive. Follow up with neck ultrasound and chest x-ray is essential to ensure complete recovery after completion of antitubercular regimen.

The work has been submitted in line with the SCARE criteria [9].

## CRediT authorship contribution statement

Concept and design: Shahbaz Habib Faridi, Md Nafees Ahamad, Syed Hasan Harris.

Acquisition, analysis and interpretation of data: Areeba Khursheed, Ezaz Ahmed, Bushra Siddiqui.

Drafting of the manuscript: Areeba Khursheed, Ezaz Ahmed.

Critical review of the manuscript: Shahbaz Habib Faridi, Bushra Siddiqui.

Supervision: Shahbaz Habib Faridi, Md Nafees Ahamad, Syed Hasan Harris.

## Consent

Written consent was taken from the patient and may be obtained as per requirement.

## Ethical approval

Ethical approval was exempted from the ethical committee.

## Guarantor

I, Areeba Khursheed, corresponding author of the case report take full responsibility for the work and conduct of the study.

## Funding

No funding procured.

## Research registration

Not applicable.

## Declaration of competing interest

The authors have declared that no competing interests exist.
